# Lactic Acid Bacteria from Kefir Increase Cytotoxicity of Natural Killer Cells to Tumor Cells

**DOI:** 10.3390/foods7040048

**Published:** 2018-03-27

**Authors:** Takuya Yamane, Tatsuji Sakamoto, Takenori Nakagaki, Yoshihisa Nakano

**Affiliations:** 1 Center for Research and Development Bioresources, Organization for Research Promotion, Osaka Prefecture University, Sakai, Osaka 599-8570, Japan; sakamoto@biochem.osakafu-u.ac.jp (T.S.); nakano@biochem.osakafu-u.ac.jp (Y.N.); 2 Department of Applied Life Sciences, Graduate School of Life and Environmental Sciences, Osaka Prefecture University, Sakai, Osaka 599-8531, Japan; 3 Institute of Food Sciences, Nakagaki Consulting Engineer and Co., Ltd., Nishi-ku, Sakai 593-8328, Japan; tnakagaki@nakagaki.co.jp

**Keywords:** kefir, lactic acid bacteria, natural killer cell, cytotoxicity

## Abstract

The Japanese fermented beverage, homemade kefir, contains six lactic acid bacteria: *Lactococcus. lactis* subsp. *Lactis*, *Lactococcus*. *lactis* subsp. *Cremoris*, *Lactococcus. Lactis* subsp. *Lactis biovar diacetylactis*, *Lactobacillus plantarum*, *Leuconostoc meseuteroides* subsp. *Cremoris* and *Lactobacillus casei*. In this study, we found that a mixture of the six lactic acid bacteria from kefir increased the cytotoxicity of human natural killer KHYG-1 cells to human chronic myelogenous leukemia K562 cells and colorectal tumor HCT116 cells. Furthermore, levels of mRNA expression and secretion of IFN-γ (interferon gamma) increased in KHYG-1 cells that had been treated with the six lactic acid bacteria mixture from kefir. The results suggest that the six lactic acid bacteria mixture from kefir has strong effects on natural immunity and tumor cell cytotoxicity.

## 1. Introduction

The fermented beverage kefir has many health benefits, including its antibacterial, anticarcinogenic, wound healing, antiallergenic, immunomodulation, and gastrointestinal immunity effects [1]. The fermented beverage, homemade kefir, has been available for more than 20 years in Japan. Homemade kefir contains six lactic acid bacteria: *Lactococcus. lactis* subsp. *Lactis* [2,3,4,5,6,7,8,9,10,11,12], *Lactococcus. lactis* subsp. *Cremoris* [8,9,13], *Lactococcus. Lactis* subsp. *Lactis biovar diacetylactis* [4], *Lacto bacillus planterun* [4,14,15,16], *Leuconostoc meseuteroides* subsp. *Cremoris* [7,9], and *Lacto bacillus casei* [2,5,12,16]. Kefir is able to offer health benefits through modulation of the gastrointestinal immune system, and previous studies showed that kefir increases the levels of some proinflammatory cytokines including TNF-α (tumor necrosis factor alpha), IFN-γ (interferon gamma) and IL-12 (interleukin 12) [1]. Lactic acid bacteria have beneficial effects with respect to diarrhea [17,18], food allergies [19], and inflammatory bowel disease [20,21,22]. Lactic acid bacteria also play an important role in the prevention of colorectal cancer [23,24], and *Lacto bacillus casei* Shirota increases the activity of natural killer (NK) cells [25,26]. However, the effect of homemade kefir on NK cell activity is not clear. In this study, we found that a six lactic acid bacteria mixture from homemade kefir increases NK cell activity, including cytotoxic activity against human colorectal tumor HCT116 cells. These results suggest that the mixture of six lactic acid bacteria from homemade kefir has important effects on innate immunity and tumor cell cytotoxicity.

## 2. Materials and Methods

### 2.1. Materials

Homemade kefir was provided by Nakagaki Consulting Engineer and Co., Ltd. (Osaka, Japan). Roswell Park Memorial Institute (RPMI) 1640 medium, Dulbecco’s modified Eagle’s medium (DMEM), and human interleukin-2 were purchased from Wako Pure Chemical Industries, Ltd. (Osaka, Japan). All other reagents were of analytical grade.

### 2.2. Cell Culture

KHYG-1 and K562 cells were provided by JCRB Cell Bank (Osaka, Japan), and HCT116 cells were purchased from American Tissue Culture Collection (ATCC) (Manassas, VA, USA). KHYG-1 cells were cultured in RPMI 1640 medium with 10% fetal bovine serum (Sigma, St. Louis, MO, USA) and 1 μg/mL IL-2. K562 and HCT116 cells were cultured in RPMI 1640 medium and in Dulbecco’s modified Eagle’s medium (DMEM), respectively, with 10% fetal bovine serum.

### 2.3. Preparation of Lactic Acid Bacteria Mixture

One gram of homemade kefir powder was added to 1 L of de Man, Rogosa and Sharpe (MRS) broth and cultured for 24 h at 22.5 °C. The cultured solution was centrifuged at 3000 rpm for 30 min at 4 °C using a TOMY centrifugation apparatus (TOMY SEIKO, Tokyo, Japan), and the six lactic acid bacteria mixture was obtained from the precipitates. A lactic acid bacteria mixture from killed bacteria was prepared by autoclaving at 120 °C for 15 min. Identification of the lactic acid bacteria cultured in the MRS broth was performed by polymerase chain reaction (PCR) using DNA from the six lactic acid bacteria as templates. After PCR, amplified DNA was separated on 2.0% agarose gels and stained with ethidium bromide. The PCR conditions and primers used are shown in Table 1.

### 2.4. Measurement of NK Cell Activity and Tumor Cytotoxicity

KHYG-1 cells were treated with the six lactic acid bacteria mixture for 24 h at 37 °C in a 5% CO_2_ incubator. The cells that had been treated with the six lactic acid bacteria mixture were reacted with K562 cells or with HCT116 cells for 4 h at 37 °C in a 5% CO_2_ incubator. NK cell activity and tumor cytotoxicity were measured using an LDH Cytotoxicity Detection Kit (Takara, Shiga, Japan).

### 2.5. RT-PCR (Reverse Transcription Polymerase Chain Reaction)

Nucleotide sequences of primers used for RT-PCR were as follows: hIFNγ-F: 5′-GAATGTCCAACGCAAAGCAA-3′, hIFNγ-R: 5′-GCTGCTGGCGACAGTTCAG-3′, hACTB-F: 5′-CTTCCTGGGCATGGAGTC-3′, and hACTB-R: 5′-GGATGTCCACGTCACACTTC-3′. The full RNAs were prepared from cells and subjected to semi-quantitative RT-PCR analyses. PCR conditions were 1 min at 94 °C, 30 s at 94 °C, 30 s at 60 °C, and 35 cycles of 1 min at 72 °C for IFNγ and ACTB. After the reactions, PCR products were separated on 2.0% agarose gels and stained with SAFELOOK™ Pregreen (Wako, Osaka, Japan). Intensities of the bands were quantified using Image J software (https://imagej.nih.gov/ij/). β-actin mRNA was also amplified as a control.

### 2.6. IFN-γ Measurement

IFN-γ was measured using a Human IFN-γ ELISA (enzyme-linked immunosorbent assay) Development Kit (PeproTech, Rocky Hill, NJ, USA) according to the manufacturer’s instructions.

### 2.7. Statistical Analysis

Data are expressed as means ± S.E (standard error). Statistical analyses were performed with an analysis of variance (one-way ANOVA) followed by an unpaired Student’s *t*-test.

## 3. Results and Discussion

### 3.1. Induction of NK Activity by the Six Lactic Acid Bacteria from Homemade-Kefir

**Figure 1:**
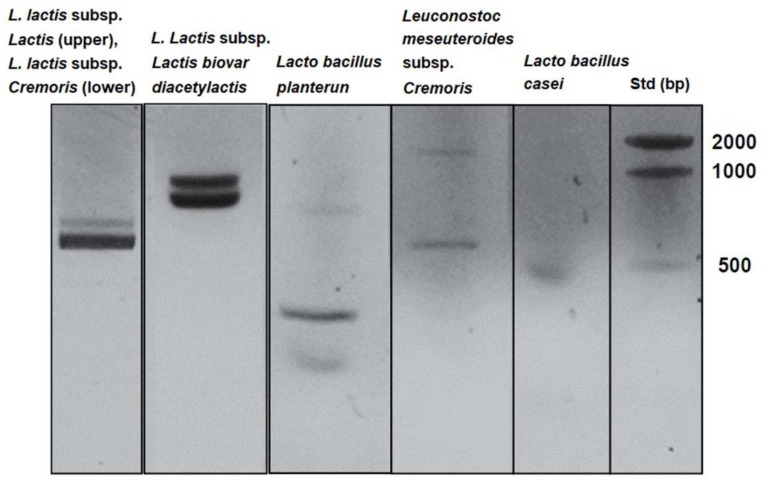
Identification of the six lactic acid bacteria cultured in de Man, Rogosa and Sharpe (MRS) broth using polymerase chain reaction (PCR) as described in the Materials and Methods. The amplified DNA after PCR were separated on agarose gels.

**Figure 2:**
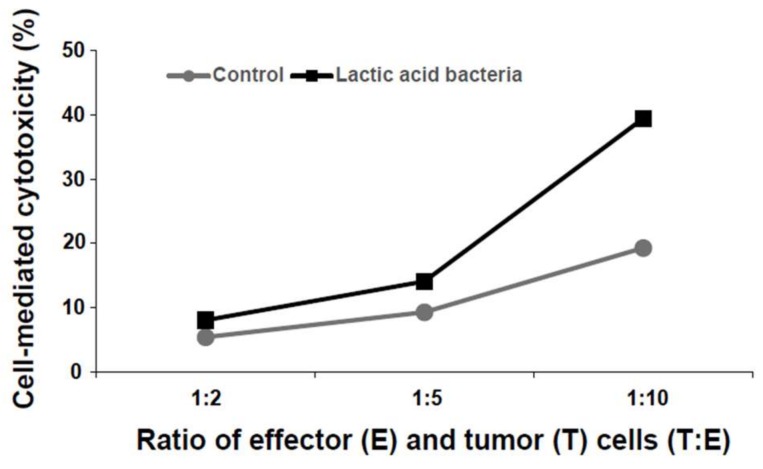
Cell-mediated cytotoxicity of natural killer (NK) cells by a six lactic acid bacteria mixture. KHYG-1 cells were treated with a six lactic acid bacteria mixture for 24 h at 37 °C in a 5% CO_2_ incubator. The treated cells were reacted with K562 cells for 4 h at 37 °C in a 5% CO_2_ incubator. Cell-mediated cytotoxicity increased for KHYG-1 cells that had been treated with the six lactic acid bacteria mixture.

**Figure 3:**
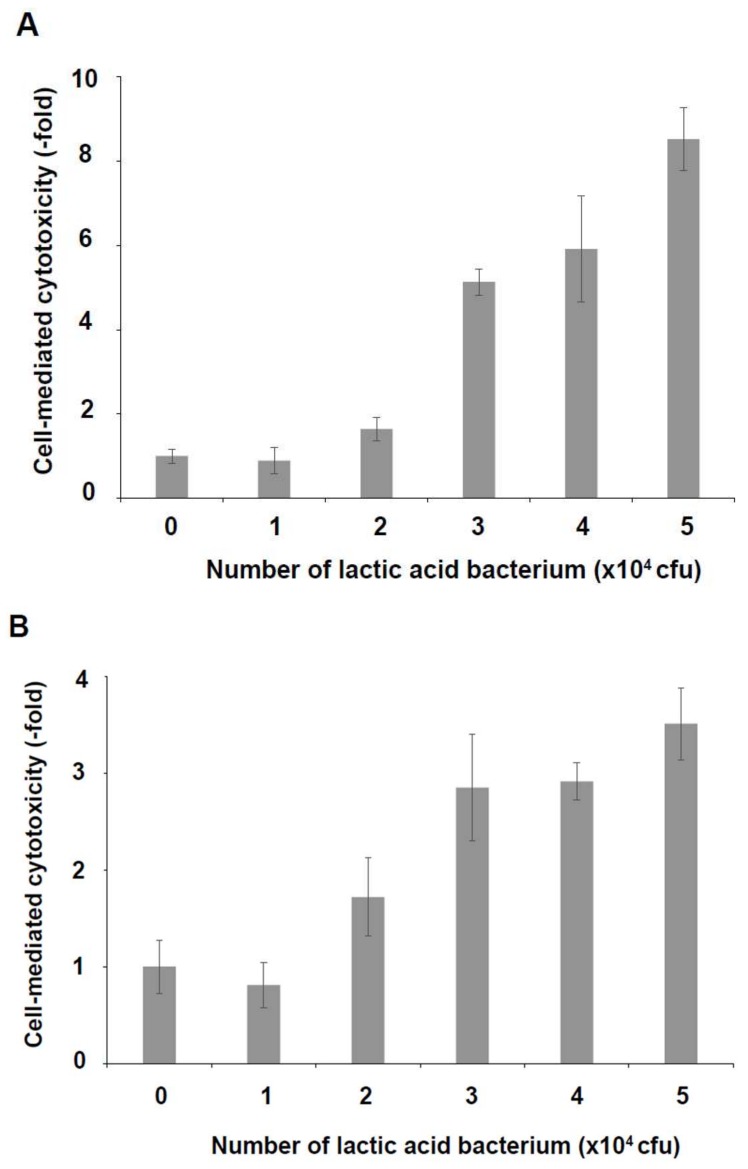
Induction of cell-mediated cytotoxicity by a six lactic acid bacteria mixture. KHYG-1 cells were treated with a six lactic acid bacteria mixture for 24 h at 37 °C in a 5% CO_2_ incubator. The treated cells were reacted with K562 cells (**A**) or with HCT116 cells (**B**) for 4 h at 37 °C in a 5% CO_2_ incubator. In both cases, cell-mediated cytotoxicity was increased by KHYG-1 cells that had been treated with the six lactic acid bacteria mixture in a dose-dependent manner.

As shown in Figure 1, the six lactic acid bacteria from homemade kefir were detected in MRS broth cultures and identified using PCR with the bacteria’s DNA as templates. The cytotoxicity of KHYG-1 cells that had been treated with the six lactic acid bacteria mixture, against K562 cells, increased in a cell number-dependent manner (Figure 2). As shown in Figure 3A, KHYG-1 cells were activated by the six lactic acid bacteria mixture and increased their cytotoxicity against K562 cells in a dose-dependent manner. Lactic acid bacteria have many effects on biological activities such as apoptosis induction, antioxidant activities, and immune response improvement [33]. Nagao et al. reported that *Lactobacillus casei Shirota* enhanced NK cell activity [25]. *Lacto bacillus plantarum C4* isolated from kefir has also been shown to prevent infection with *Yersinia enterocolitice* O9 in the intestine in mice. Additionally, a concentrated supernatant from the cultured medium of *Lacto bacillus plantarum C4* has antibacterial and anti-tumor activities [34]. Since the six lactic acid bacteria mixture from homemade kefir includes *Lacto bacillus plantarum* and *Lacto bacillus casei*, it is thought that these lactic acid bacteria contribute to the induction of NK cell activity.

### 3.2. Induction of Cytotoxicity by the Six Lactic Acid Bacteria Against Human Colorectal Tumor Cells

Cytotoxic activity of KHYG-1 cells against HCT116 cells was increased by the six lactic acid bacteria mixture in a dose-dependent manner (Figure 3B). NK cells have the intrinsic ability to detect and kill cancer cells [35]. The lytic functions of NK cells mediated by granzyme B and perforin have dominated investigations of the role of NK cells in anti-cancer defense [36]. The effector function of NK cells is tightly regulated by activating and inhibiting receptors. Tumor cells can evade the anti-tumor activity of NK cells by modifying the expression of NK cell receptors. In colorectal carcinoma, for instance, the expression of activating receptors—such as CD16 (FCGR3A, Fc fragment of IgG receptor IIIa), NKp46 (NCR1, natural cytotoxicity triggering receptor 1), NKp30 (NCR3, natural cytotoxicity triggering receptor 3), and NKG2D (KLRK1, killer cell lectin like receptor K1)—is decreased. Additionally, the expression of inhibiting receptors, such as KIR3DL1 (killer cell immunoglobulin-like receptor 3DL1) and NKG2A (killer cell lectin like receptor C1), is increased [37]. The six lactic acid bacteria mixture or its secretory compounds may regulate the expression of these receptor genes and inhibit tumor formation by escaping from NK cell-mediated cytotoxicity. Lactic acid bacteria have indeed been demonstrated to have a host of properties for preventing the development of a colorectal tumor, specifically by inhibiting its initiation or progression. In the pathway of the anticancer immune response, lactic acid bacteria also stimulate NK cell activity [33]. Furthermore, a seminal 11-year prospective cohort study conducted in Japan showed an inverse correlation between increase of cancer incidence and the level of NK cell cytotoxicity [38]. These results indicate that the six lactic acid bacteria mixture from homemade kefir reacts with human colorectal tumor HCT116 cells through activation of the immunity of NK cells.

### 3.3. Induction of Cytotoxicity of KHYG-1 Cells by the Killed Lactic Acid Bacteria Mixture

**Figure 4:**
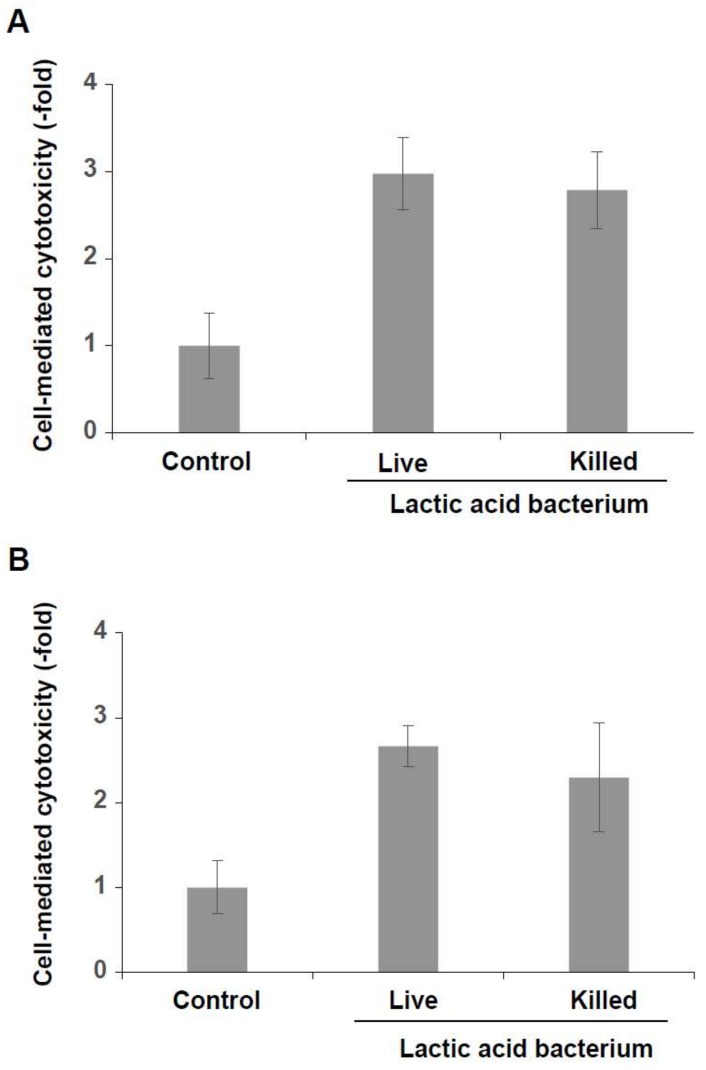
Induction of cell-mediated cytotoxicity of KHYG-1 cells by the live and the killed lactic acid bacteria mixtures. KHYG-1 cells were treated with both mixtures (3 × 10^4^ cfu) for 24 h at 37 °C in a 5% CO_2_ incubator. The treated cells were reacted with K562 cells (**A**) or with HCT116 cells (**B**) for 4 h at 37 °C in a 5% CO_2_ incubator. In both cases, cell-mediated cytotoxicity was increased in KHYG-1 cells that had been treated with both the live and the killed lactic acid bacteria mixtures.

To examine the distinct effects of live and dead lactic acid bacteria on NK cell activation, KHYG-1 cells were treated with the live and the killed six lactic acid bacteria mixtures and cytotoxicity assays were carried out. As shown in Figure 4A, activation of KHYG-1 cells was increased by treatment with both the live and killed mixtures. Cytotoxicity of KHYG-1 cells against HCT116 cells was also increased by both mixtures (Figure 4B). Previous studies, on the other hand, have shown that 40% to 62% of several strains of *L. plantarum* survive under the low pH conditions in a simulated intestine [39], and that 81% to 93% of several strains of *L. casei* can also survive at pH 2.0 for 2 h [40]. The present and previous results, therefore, suggest that both lactic acid bacteria that have been killed by gastric acid and live lactic acid bacteria have effects on NK cell activation in the intestine after the administration of homemade kefir.

### 3.4. Increased Expression and Secretion Levels of IFN-γ in KHYG-1 Cells

**Figure 5:**
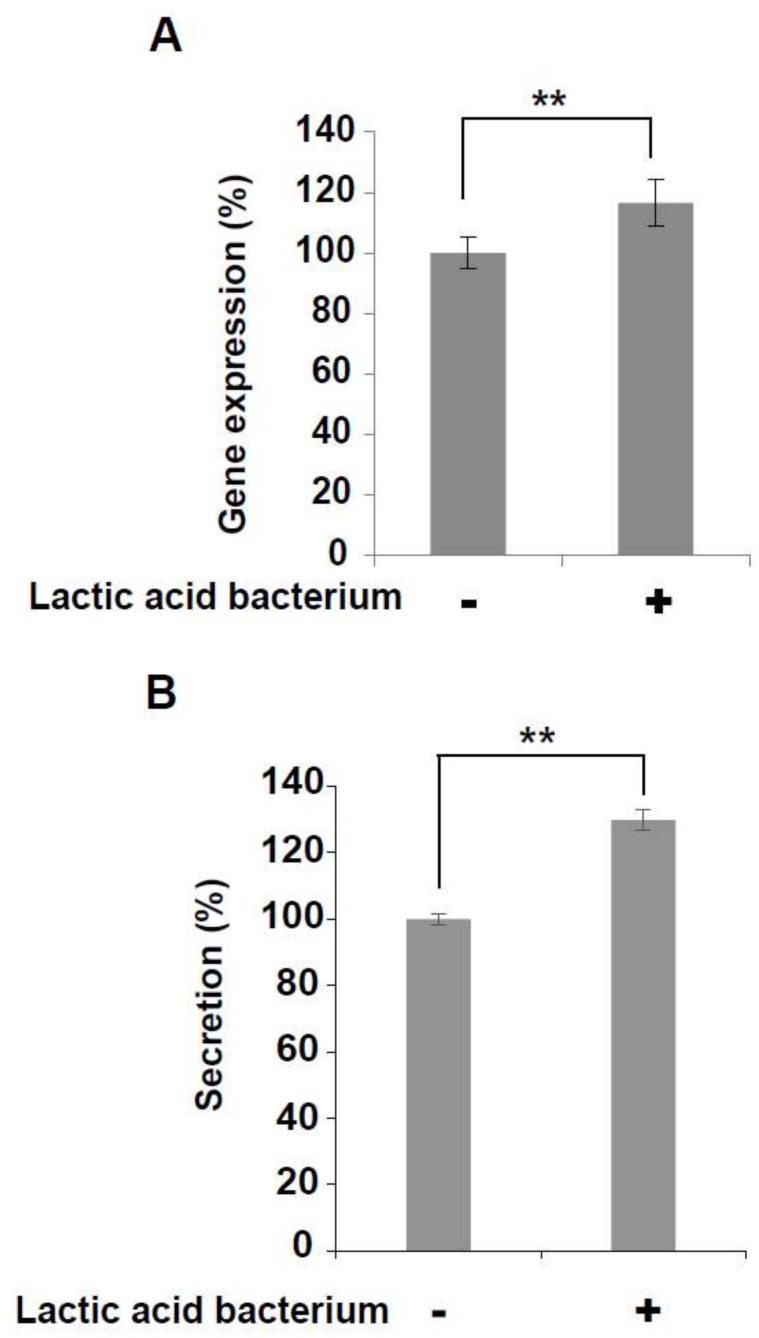
Increased expression and secretion levels of IFN-γ. (**A**) The expression level of IFN-γ mRNA was increased in KHYG-1 cells that had been treated with the six lactic acid bacteria mixture (3 × 10^4^ cfu) for 4 h at 37 °C in a 5% CO_2_ incubator (*n* = 3, ** *p* < 0.01). (**B**) The secretion level of IFN-γ mRNA was increased in the same, treated, KHYG-1 cells (*n* = 6, ** *p* < 0.01).

IFN-γ mRNA and IFN-γ secretion were both increased in KHYG-1 cells that had been treated by the six lactic acid bacteria mixture (Figure 5A,B). NK cells regulate various immune cells through the secretion of IFN-γ and they perform tumor surveillance [41]. Increased IFN-γ expression and secretion induced by the mixture of six lactic acid bacteria from homemade kefir may provide a synergistic effect on anti-tumor activity.

## 4. Conclusions

This study showed that the cytotoxicity of NK cells and the expression and secretion of IFN-γ in NK cells were increased after treatment of the cells with a six lactic acid bacteria mixture. Further in vitro and in vivo studies using animal models of colorectal cancer are needed to investigate the mechanisms underlying the effects of the six lactic acid bacteria mixture.

## Figures and Tables

**Table 1:** Polymerase chain reaction (PCR) primers and reacting conditions.

Strain	Primer Sequence		PCR Condition	Ref.
*L. lactis* subsp. *Lactis*	sense	5′-CGTTATGGATTTGATGGATATAAAGC-3′	94 °C 9 min, 94 °C 30 s, 50 °C 30 s, 72 °C 60 s × 45 cycles, 72 °C 7 min	[27]
	antisense	5′-ACTCTTCTTAAGAACAAGTTTAACAGC-3′		
*L. lactis* subsp. *Cremoris*	sense	5′-CGTTATGGATTTGATGGATATAAAGC-3′	94 °C 9 min, 94 °C 30 s, 50 °C 30 s, 72 °C 60 s × 45 cycles, 72 °C 7 min	
	antisense	5′-ACTCTTCTTAAGAACAAGTTTAACAGC-3′		
*L. lactis* subsp. *lactis biovar diacetylactis*	sense	5′-CTTCGTTATGATTTTACA-3′	94 °C 30 s, 94 °C 30 s, 46 °C 60 s, 72 °C 60 s × 30 cycles, 72 °C 2 min	[28,29]
	antisense	5′-AATATCAACAATTCCATG-3′		
*Leuconostoc mesenteroides* subsp. *Cremoris* (Genus-specific primers for *Leuconostoc*)	sense	5′-AACTTAGTGTCGCATGAC-3′	94 °C 5 min, 94 °C 60 s, 60 °C 60 s, 72 °C 60 s × 30 cycles, 72 °C 10 min	[30]
	antisense	5′-AGTCGAGTTACAGACTACAA-3′		
*Lactobacillus plantarum*	sense	5′-CCGTTTATGCGGAACACCTA-3′	94 °C 3 min, 94 °C 30 s, 54 °C 10 s, 72 °C 30 s × 30 cycles, 72 °C 5 min	[31]
	antisense	5′-TCGGGATTACCAAACATCAC-3′		
*Lactobacillus casei*	sense	5-TGCACTGAGATTCGACTTAA-3	94 °C 3 min, 94 °C 45 s, 45 °C 45 s, 72 °C 60 s × 30 cycles, 72 °C 5 min	[32]
